# Role of Namaste Care: A Person-Centered Approach to Enhance the Quality of Life of the Elderly with Alzheimer’s Disease

**DOI:** 10.31661/gmj.v12i.3205

**Published:** 2023-12-18

**Authors:** Rafat Rezapour-Nasrabad, Marzieh Heydari, Seyedeh Fatemeh Moosavi Moqaddam, Saeedeh Piri, Elham Sadeghi Moghimi, Sanaz Rustaee

**Affiliations:** ^1^ Department of Psychiatric Nursing and Management, School of Nursing and Midwifery, Shahid Beheshti University of Medical Sciences, Tehran, Iran; ^2^ Department of Educational Sciences, Abadeh Branch, Islamic Azad University, Abadeh, Iran; ^3^ Department of Nursing, School of Nursing, Jahrom University of Medical Sciences, Jahrom, Iran; ^4^ Department of Nursing and Midwifery, Social Development and Health Promotion Research Center, Health Institute, Kermanshah University of Medical Sciences, Kermanshah, Iran; ^5^ Department of Community Health Nursing, Vali Asr Nursery, Shiraz, Iran; ^6^ Department of Nursing Internal Surgery, School of Nursing, Fasa University of Medical Sciences, Fasa, Iran

**Keywords:** Namaste Care, Alzheimer’s Disease, Elderly, Quality of Life

## Dear Editor,

**Table:** Table1. Some Important Studies On the Role of Namaste Care On the Quality of Life of Elderly Individuals with Alzheimer’s Disease

Study	Participants	Intervention(s)	Outcomes
Zeisel et al. (2003) [7]	193 residents with AD in special care units	Implementation of environmental modifications, including NC	↑QoL ↓Agitation ↑Engagement in activities
van der Ploeg et al. (2013) [8]	14 nursing home residents	Sensory stimulation, music therapy, and massage	↑QoL ↓Agitation ↑Engagement in activities
Stacpoole et al. (2017) [9]	37 nursing home residents	Sensory stimulation, music therapy, and massage	↑QoL
McMurdo et al. (2014) [10]	27 nursing home residents	Sensory stimulation, music therapy, and massage	↑QoL ↓Agitation ↑Engagement in activities
Smaling et al. (2018) [11]	16 nursing home residents and their family members	Sensory stimulation, music therapy, and massage	↑QoL ↓Discomfort ↑Pleasure
Van Haitsma et al. (2013) [12]	1,003 community-dwelling elders	PELI*	↑Life satisfaction ↓Depression
van der Steen et al. (2018) [13]	128 nursing home residents	Sensory stimulation, music therapy, and massage	↑QoL ↓Agitation ↑Engagement in activities
Amrollah Majdabadi Kohne et al. (2021) [14]	25 nursing home residents	Implemented NC for two hours daily and four days per week for six months	↑QoL

^*^ Includes items related to sensory stimulation and social interaction **NC:** Namaste care; **AD:** Alzheimer’s disease; **QoL:** Quality of life; **PELI:** Preferences for everyday living inventory

Alzheimer’s disease (AD) is a progressive neurodegenerative disorder that affects millions of people worldwide [1]. It is a devastating disease that not only affects the individual but also their families and caregivers [2]. Namaste Care (NC) is a holistic approach to care for individuals with advanced dementia, including AD [3]. It was first introduced in the United Kingdom in the early 2000s as a way to provide personalized and compassionate care for those who are no longer able to communicate or participate in traditional activities [4]. The approach is based on the principles of person-centered care, which emphasizes the importance of treating each individual as a unique person with their own preferences, needs, and values [4]. Indeed, NC is designed to provide comfort and support to individuals with advanced dementia by engaging their senses and creating a calming and soothing environment [5]. It involves a range of activities, such as gentle massage, aromatherapy, music therapy, and sensory stimulation [6]. The primary aim of NC is to create a peaceful and relaxing environment that promotes a sense of well-being and reduces agitation and anxiety [6].

As shown in Table-1, previous studies [7][8][9][10][11][12][13][14] revealed that NC effectively reduced agitation as well as enhanced QoL.

In a pilot study, we evaluated the role of NC on the QoL of elderly patients with AD who were residents in a nursing home in Tehran. Briefly, 25 patients including 16 women were selected and the QoL was measured using the Persian version of the QoL in late-stage dementia (QUALID) questionnaire, and after received four months of NC by a trained caregiver, it was re-evaluated. Our finding indicated that NC could significantly improve the QoL of patients with AD in line with previous evidence.

Compared to other treatment options for individuals with advanced dementia, NC has several unique advantages. Unlike medication-based treatments, NC does not have any negative side effects and is not associated with the risk of drug interactions or complications [15]. Additionally, NC is a non-invasive and non-pharmacological approach that can be easily adapted to the individual needs and preferences of each person.

Overall, studies suggest that NC can have a positive impact on the QoL of elderly individuals with AD, which can lead to improved well-being. However, most of these studies have small sample sizes and are performed on one gender, which may not be generalizable to all individuals with AD. Hence, further research with a larger sample size as well as longer follow-up are needed to confirm the effectiveness of NC and determine the optimal components of the intervention.

## Conflict of Interest

All the authors declare there are no competing interests.
